# Cerebrospinal fluid leak presented with the C1-C2 sign caused by spinal canal stenosis: a case report

**DOI:** 10.1186/s12883-020-01697-1

**Published:** 2020-04-23

**Authors:** Chihiro Akiba, Hideki Bandai, Yoshitaka Ito, Tsuyoshi Maeda, Keisuke Yamaguchi, Madoka Nakajima, Masakazu Miyajima

**Affiliations:** 1 Department of Neurosurgery, Juntendo Tokyo Koto Geriatric Medical Center, 3-3-20 Shinsuna, Koto-ku, Tokyo, 136-0075 Japan; 2 Department of Anesthesiology, Juntendo Tokyo Koto Geriatric Medical Center, 3-3-20 Shinsuna, Koto-ku, Tokyo, 136-0075 Japan, Japan

**Keywords:** Case report, Cerebrospinal fluid leak, C1-C2 sign, Intracranial hypotension, Spinal canal stenosis

## Abstract

**Background:**

Intracranial hypotension is a disorder characterized by low cerebrospinal fluid (CSF) pressure typically caused by loss of CSF. Although some mechanisms account for the CSF leakage have been elucidated, spinal canal stenosis has never been reported as a pathological cause of intracranial hypotension.

C1-C2 sign is a characteristic imaging feature, which indicates CSF collection between the spinous processes of C1 and C2, occasionally observed on magnetic resonance imaging (MRI) in patients with intracranial hypotension.

**Case presentation:**

A 58-year-old man was presented to our institute with complaints of posterior cervical pain persisting for 3 months, along with numbness and muscle weakness of extremities. A fat suppression T2-weighted image of MRI illustrated fluid collection in the retrospinal region at C1-C2 level, and an 111In-DTPA cisternoscintigram clearly revealed the presence of CSF leakage into the same region. The MRI also showed stenosis in spinal canal at C3/4 level, and a computed tomography (CT) myelogram suggested a blockage at the same level. We gave a diagnosis as intracranial hypotension due to the CSF leakage, which might be caused by the spinal canal stenosis at C3/4 level. Despite 72 h of conservative therapy, a brain CT showed the development of bilateral subdural hematomas. We, therefore, performed burr-hole drainage of the subdural hematoma, blood-patch therapy at C1/2 level, and laminoplasty at C3–4 at the same time. Improvement of symptoms and imaging features which suggested the CSF leak and subdural hematoma were obtained post-operatively.

**Conclusion:**

The present case suggested the mechanism where the CSF leakage was revealed as fluid collection in the retrospinal region at C1-C2 level. Increased intradural pressure due to the spinal canal stenosis resulted in dural tear. CSF leaked into the epidural space and subsequently to the retrospinal region at C1-C2 level, due to the presence of spinal canal stenosis caudally as well as the vulnerability of the tissue structure in the retrospinal region at C1-C2 level. Thus, our theory supports the mechanisms of previously reported CSF dynamics associated to C1-C2 sign, and also, we suggest spinal canal stenosis as a novel etiology of intracranial hypotension.

## Background

Intracranial hypotension has gained increasing recognition over the past 20 years [1–4], as a disorder of low cerebrospinal fluid (CSF) pressure characterized by postural headache, brain sagging and chronic subdural hematomas [5–7]. Intracranial hypotension is typically caused by loss of CSF through nerve root sleeve diverticulum, dural defect due to osteophyte spur or CSF-venous fistula in the thoracic or lumbar spine; however, underlying pathophysiology remains incompletely understood [4]. Further, to the best of our knowledge, spinal canal stenosis has never been reported as a pathological cause of intracranial hypotension.

C1-C2 sign is a characteristic imaging feature, which indicates CSF collection in retrospinal soft tissue between the spinous processes of C1 and C2 levels, occasionally observed on magnetic resonance imaging (MRI) in patients with intracranial hypotension [8–10]. An interesting and important consideration of this sign is that the retrospinal point at C1-C2 level exactly indicates the site of CSF leakage (i.e. the site of dural tear) in some patients [11, 12], but not necessarily indicates the site of CSF leakage (i.e. the site of dural tear) in other patients [8, 13]. This discrepancy comes from dynamics of the leaked CSF in the spinal canal. In other words, the site of dural tear which makes the connection between intra- and epidural spaces is possibly different from the site of tear on the posterior atlantoaxial membrane at C1-C2 level which makes fluid collection in the retrospinal tissue.

We have recently encountered a patient with intracranial hypotension in association with spinal canal stenosis as its potential etiological factor, and were presented with a characteristic imaging with the C1-C2 sign. Moreover, we performed combined therapies which include burr-hole drainage of the subdural hematoma, blood-patch therapy and laminoplasty. Here, we report suggestive strategies of diagnosis and treatment of intracranial hypotension.

## Case presentation

**Fig. 1:**
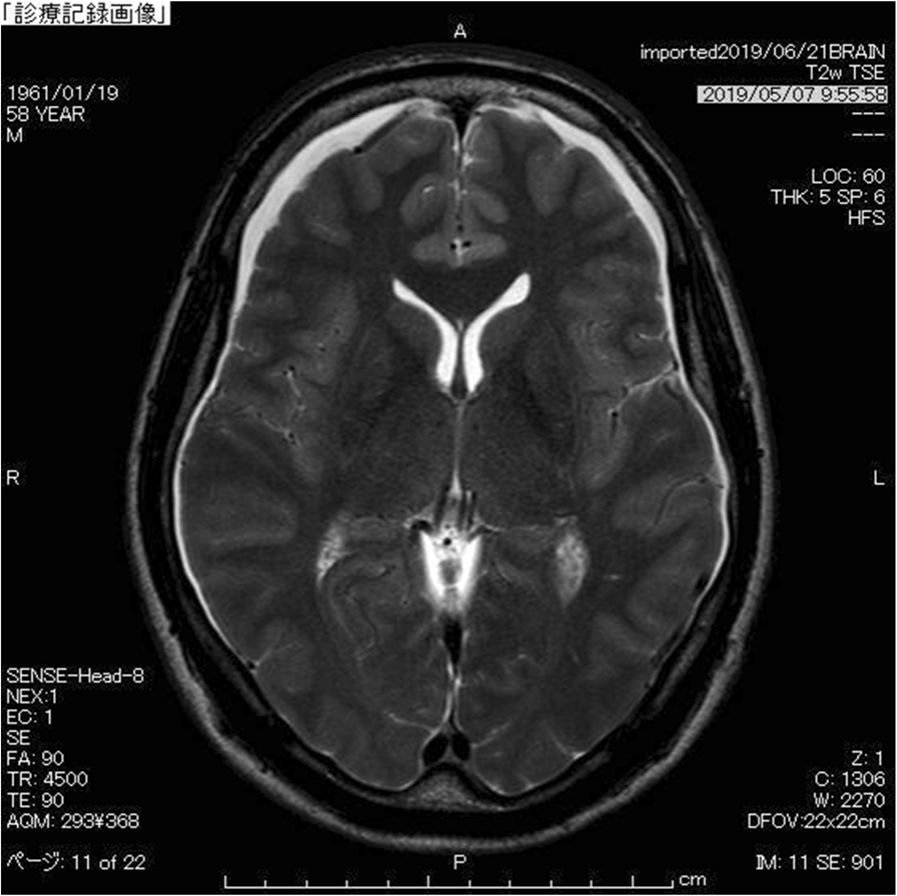
Subdural effusion at the onse. Bilateral subdural effusion was demonstrated by T2-weighted image of brain magnetic resonance imaging (MRI)

A 58-year-old Japanese man was presented to our out-patient unit complaining of posterior cervical pain. The pain firstly appeared 3 months before the first contact to us without any particular triggers such as trauma. He was relieved from the pain when lying flat; however, he kept working as a plumber and the pain got worse over the time period. A brain computed tomography (CT) was performed 1.5 months after the onset in another clinic and it revealed bilateral subdural effusion (Fig. 1), still, no treatment was given at this point. Later on, when he was presented to our hospital, numbness and weakness of extremities also appeared in addition to the progressive posterior cervical pain as well as headache. He had a medical history of hemorrhoid and no particular familial history.

The patient admitted to our department soon after the initial contact. A hand-held dynamometer revealed his weakened grip strength of both of his hands by 30–40 kg. He was also presented with bilateral muscle weakness in his lower extremities which was 4 of 5 in the Manual Muscle Test (MMT) score [14], as well as sensory disturbances in his forearms, hands and the posterior surface of thighs on both sides. His Japanese Orthopedic Association (JOA) score [15] was 13.5.

**Fig. 2:**
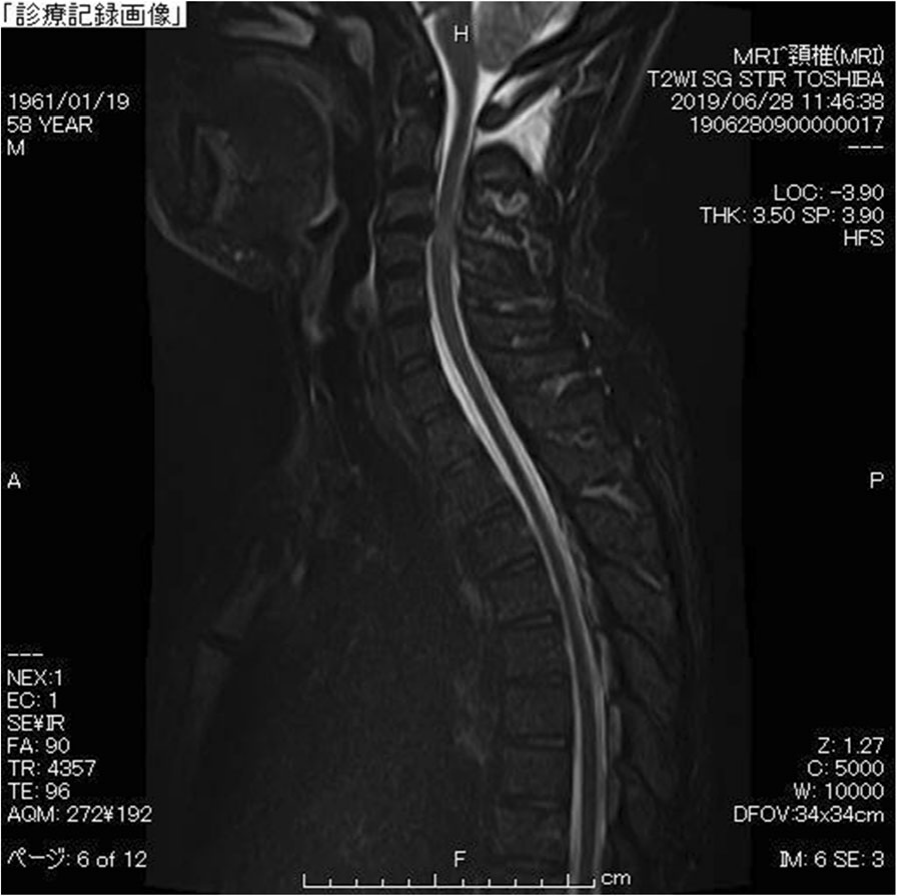
C1-C2 sign. A saggital view of T2-weighted fat suppression image of magnetic resonance imaging (MRI) in the cervical level revealed fluid collection in the retrospinal region at C1-C2 level. Additionally, a spinal canal stenosis at C3/4 level was also demonstrated

**Fig. 3:**
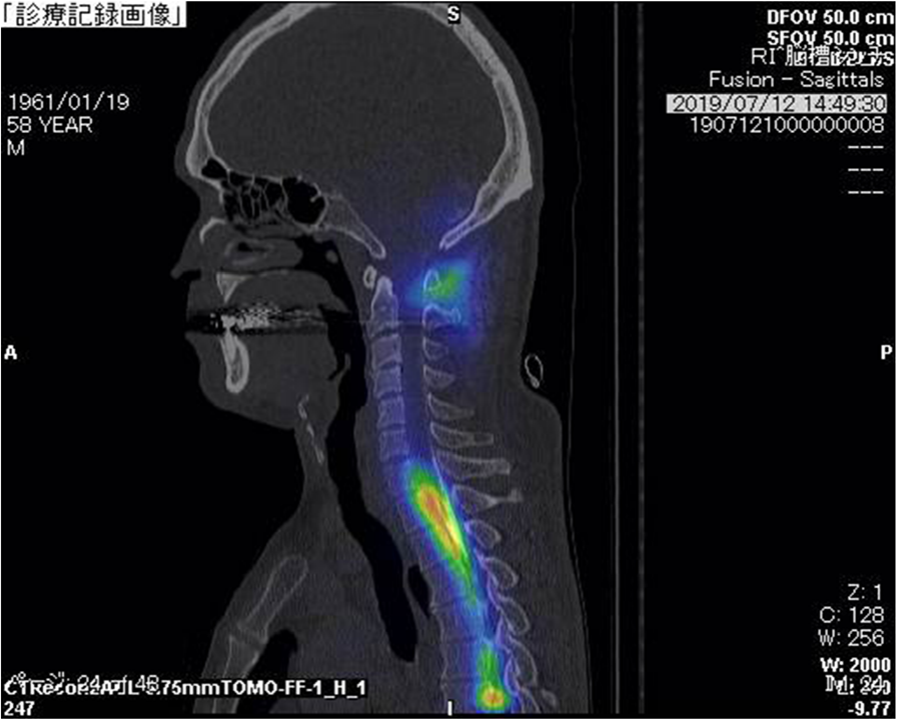
CSF leakage detected by cisternoscintigram. A saggital view of cisternoscintigram in the cervical level demonstrated CSF leak in the retrospinal region at C1-C2 level

**Fig. 4:**
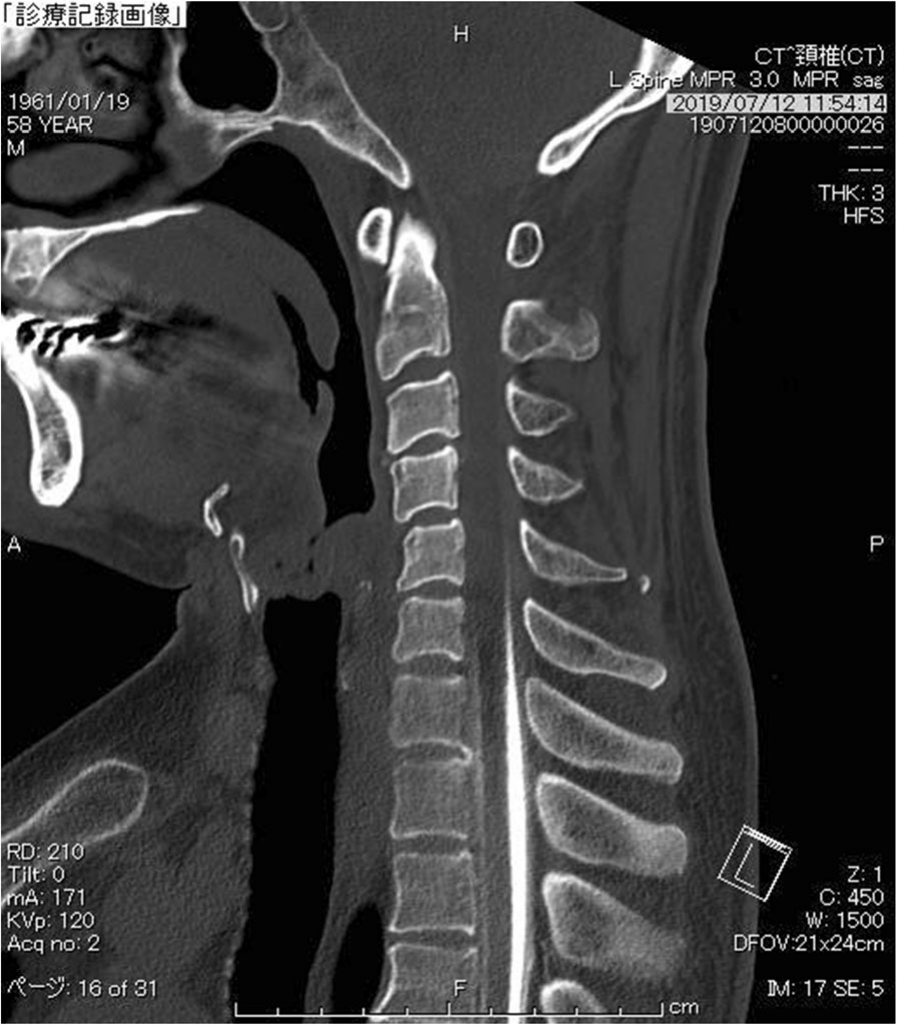
CSF blockage detected by computed tomography (CT) myelogram. A saggital view of CT myelogram illustrated CSF blockage due to the spinal canal stenosis at C3/4 level. On the other hand, no sign of CSF leak was demonstrated

A fat suppression T2-weighted MRI without gadolinium enhancement of the cervical spine illustrated a fluid collection in the soft tissue spaces of the retrospinal region at C1-C2 level (Fig. 2). Additionally, it demonstrated spinal canal stenosis at C3/4 level. Subsequently, a cisternoscintigram and a CT myelogram were performed with intradural injection of 111In-DTPA (Nihon Mediphysics, Tokyo, Japan) and Omnipaque 240® (Daiichi-Sankyo, Tokyo, Japan), an iodine contrast agent, through a lumber puncture. The 111In-DTPA cisternoscintigram clearly revealed CSF leakage into the retrospinal region at C1-C2 level, as well as blockage of the 111In-DTPA at lower level of the cervical spinal canal (Fig. 3). Furthermore, the CT myelogram also suggested blockage at lower level of the cervical spinal canal; though it did not demonstrated the sign of CSF at C1-C2 level or any other sites (Fig. 4).

**Fig. 5:**
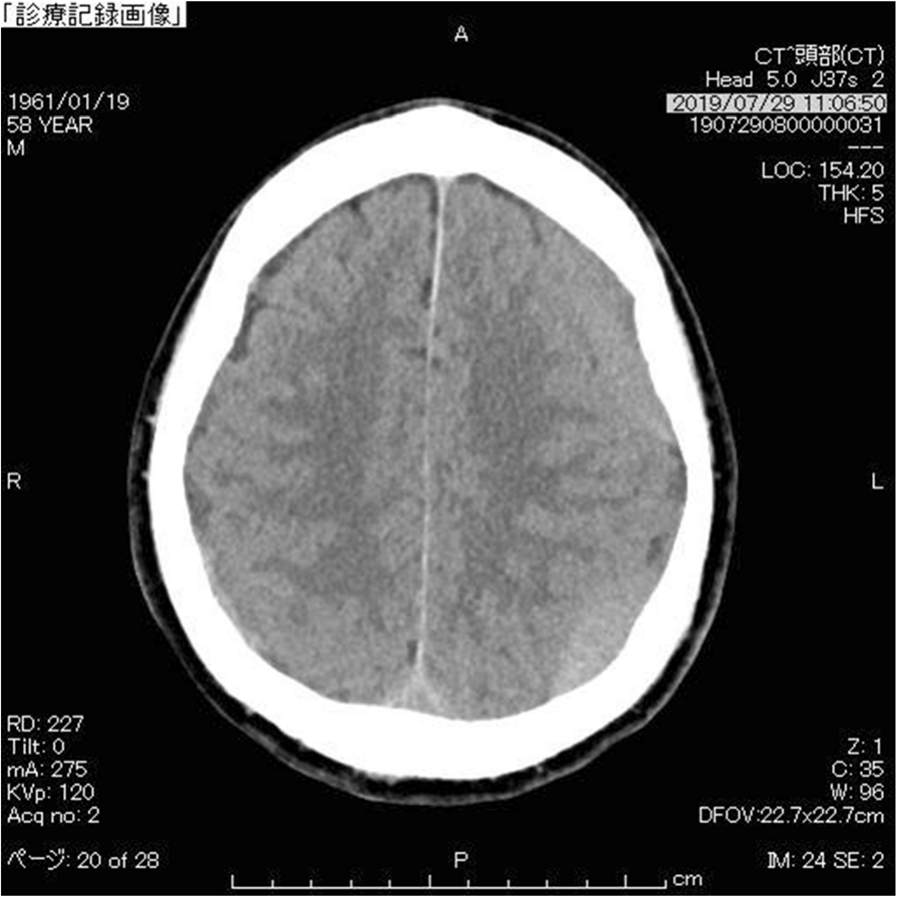
Bilateral subdural hematoma. Subdural effusion progressed to the bilateral subdural hematoma

72 h of conservative therapy, including bed rest, intravenous fluid hydration, muscle relaxants and non-steroidal anti-inflammatory drugs, was ineffective at improving his symptoms, and the re-examination of brain CT showed progression of the bilateral subdural effusions to bilateral subdural hematomas (Fig. 5).

After reviewing of his imaging data and confirmation that no other cause presented in development of subdural hematoma along with careful discussions, we gave a diagnosis of intracranial hypotension due to the CSF leakage from C1-C2 level, which might be caused by spinal canal stenosis at C3/4 level. We, therefore, performed burr-hole drainage of the subdural hematoma, blood-patch therapy at C1/2 level and laminoplasty at C3–4, at the same time. In the laminoplasty, CSF flew out from the epidural space subsequently to the opening of lamina, and after sufficient decompression, the CSF flow was reduced in the operation field, without detection and restoration of dural tear (Video). In the blood-patch therapy, an epidural catheter was inserted from the surgical field at C3–4 level to C1-C2 level under x-ray fluoroscopy observation. Approximately 5 ml of autologous blood sampled from patient’s peripheral vein was injected to the epidural site at C1-C2 level and the blood-patch therapy was achieved.

**Fig. 6:**
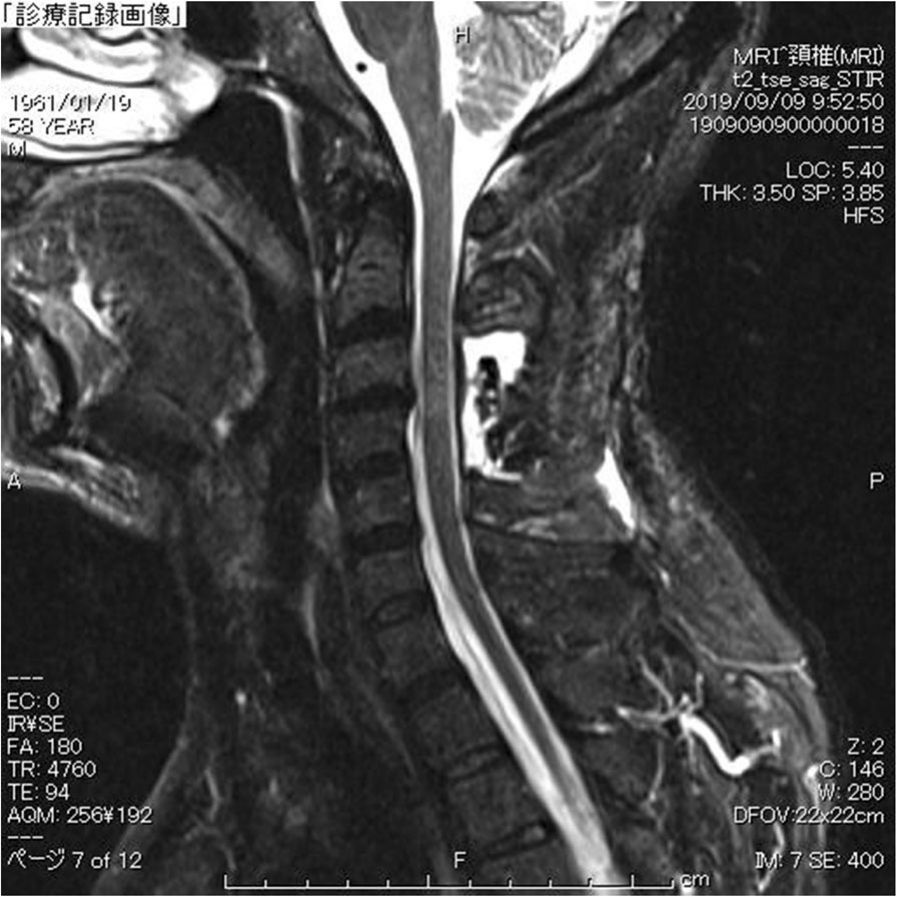
Postoperative imaging. Postoperative saggital view of T2-weighted fat-suppressed magnetic resonance imaging (MRI) in the cervical level revealed disappearing of the fluid collection in the retrospinal region at C1-C2 level. Also, the spinal canal stenosis at C3/4 level was treated


Postoperative response was significantly favorable. His symptoms completely improved and the imaging features that suggested CSF leak and intracranial hypotension disappeared (Fig. 6). He discharged on postoperative day 10 with satisfaction, and no signs of recurrence were observed for 5 months postoperatively up to this point. The patient returned to his daily life as well as his occupation.

**Additional file 1.** Microscopic pictures of the laminoplasty. Legend: On the left side of the screen indicates the cranial side and the right side indicates the caudal side. CSF flew out from the epidural space subsequently to the opening of lamina, and after sufficient decompression, the CSF flow was reduced in the operation field, without detection and restoration of dural tear.


## Discussion and conclusion

We report a case of intracranial hypotension which was potentially caused by spinal canal stenosis with C1-C2 sign on MRI fat suppression T2-weighted image. The C1-C2 sign in the present case deserves special attention. This sign is typically seen as a focal area of fluid-like signal intensity on MRI and as a CSF collection between the spinous processes of C1 and C2 on CT myelograms or a cisternoscintigrams. These imaging features are found in 12–67% of the patients with intracranial hypotension [8, 9, 13, 16]. Our imaging tests included one discrepancy where the cisternoscintigram revealed the CSF leakage at C1-C2 level, although, the CT myelogram did not. It could be because of the difference of molecular weights between 111In-DTPA and Omnipaque 240®, which were approximately 545.29 and 821.4, respectively. The smaller molecular weight allowed 111In-DTPA to go up through the blockage due to the canal stenosis, while Omnipaque 240® with bigger molecular weight was stuck.

Interestingly, though, it should be noted that C1-C2 sign does not necessarily denote the exact site of CSF leakage; hence, it has also been called as C1–C2 false localizing sign [8, 10]. Schievink et al. succeeded to reproduce the C1-C2 sign by contrasting epidural space in patients with intracranial hypotension presented with C1-C2 sign, which suggested that CSF leaked from dural damage into epidural space flew out from the spinal canal and diffusely expanded to the soft tissue of dorsal region of C1-C2. In fact, the actual leakage site reported was caudal to the fluid collections and most often originated in the lower cervical and the thoracic spine in their report. They postulated the mechanism that the enlarged epidural space due to the CSF leakage provides a capacious pathway for the leaked CSF to travel along the spinal canal, and the C1-C2 level may be the most particularly vulnerable escape site for the CSF into the soft tissues because of the absence of epidural fat, the mobility of this segment, the lack of bone support, or the loose connective tissues [8]. In support, it is reported that the posterior atlantoaxial membrane at C1-C2 level is a vulnerable site because it lacks the yellow ligament and epidural fat [17], and the retrospinal region between the myodural bridges of rectus capitis posterior major and obliquus capitis inferior are areas rich in loose connective tissues [18]. Furthermore, Klarica et al. demonstrated the cervical subarachnoid space was a site for buffering spinal CSF pressure by its elasticity, using an experimental model [19]. They showed CSF redistribution under the vertical head-down position, expanding cervical subarachnoid space, while lumber subarachnoid space was narrowed without any changes of the cranial volume. This phenomenon helps us to explain that the cervical subarachnoid space plays a role in control spinal CSF pressure by expanding and shrinking. In support, it is also suggested that the epidural lymphatics are well developed on the dorsal side of the lower cervical spinal dura mater and may function as an absorptive pathway for the CSF from the subarachnoidal space [20].

Another novel finding in the case is that spinal canal stenosis could be a pathological cause of CSF leakage and hence, intracranial hypotension. The suggested mechanism from the present case is that the spinal canal stenosis and straining could lead to increased intradural pressure which eventually resulted in the dural tear. Subsequently, the CSF leaked into the epidural space and later escaped to the retrospinal region at C1-C2 level, because of its vulnerability; and dysfunction of CSF pressure regulation in cervical subarachnoid space contributed by the obstruction of the caudal epidural space resulting from spinal canal stenosis. Thus, our theory could fit and support the mechanism of CSF dynamics in the mechanism of C1-C2 presentation advocated by Schievink et al. [8], and the capability of cervical subarachnoid space in regulating CSF pressure demonstrated by Klarica et al. [19] and Miura et al. [20]. In conclusion, reteospinal region at C1-C2 level is a frequent site of CSF collection due to the CSF leakage from any levels of the spine, because of its anatomical and functional characteristics. We also suggested the spinal canal stenosis as a novel etiology of intracranial hypotension.

## Supplementary information

**Additional file 2.**

## Data Availability

The datasets used and/or analyzed in the current study are available from the corresponding author on reasonable request.

## Abbreviations

CSF: Cerebrocspinal fluid
MRI: Magnetic resonance imaging
CT: Computed tomography
