# Supplementary material for: Cerebrospinal fluid leak presented with the C1-C2 sign caused by spinal canal stenosis: a case report
Source: BMC Neurol. 2020 Apr 23;20:151. doi: 10.1186/s12883-020-01697-1 (PMC7181568; doi:10.1186/s12883-020-01697-1)
Supplement: Supplementary file 2 — Additional file 2. [file 12883_2020_1697_MOESM2_ESM.docx]

Timeline figure

Symptom relief

Improvement of the pathological imaging features

No recurrence

X

– 3 months

posterior cervical pain

bilateral subdural effusions in CT

numbness and weakness of extremities

X

X

– 1.5 months

X

+ 3 weeks

burr-hole drainage

blood-patch therapy

laminoplasty at C3-4

MRI: C1-C2 sign &

cervical canal stenosis

Cisternoscintigram:

CSF leak at C1-C2

CT myelogram:

blockage at

cervical canal stenosis

CT: bilateral subdural

hematomas

- a plumper

- medical history of hemorrhoid
